# Comparative Study of Adductor Canal Block and Femoral Nerve Block for Postoperative Analgesia After Arthroscopic Anterior Cruciate Ligament Tear Repair Surgeries

**DOI:** 10.7759/cureus.24007

**Published:** 2022-04-10

**Authors:** Amey Dixit, Ravi Prakash, Avtar S Yadav, Sudhakar Dwivedi

**Affiliations:** 1 Department of Anaesthesiology, Shyam Shah Medical College, Rewa, IND

**Keywords:** vas score, quadriceps muscle strength, femoral nerve block, arthroscopic acl reconstruction, adductor canal block

## Abstract

Objective: The study aimed to compare an adductor canal block (ACB) with a femoral nerve block (FNB) with regard to their analgesic efficacy and the quadriceps muscle strength in patients following arthroscopic anterior cruciate ligament (ACL) tear repair surgeries.

Materials and Methods: Ninety patients in the American Society of Anaesthesiologists (ASA) status I or II undergoing arthroscopic ACL tear repair surgeries under subarachnoid block were divided into three groups to receive ACB (Group ACB), FNB (Group FNB), and control (Group C). Each patient was assessed for Visual Analogue Scale (VAS) score, tramadol consumption, and quadriceps muscle strength postoperatively in the post anaesthesia care unit (PACU).

Results: There was no significant difference between the Group ACB and Group FNB regarding postoperative analgesia and total rescue analgesic consumption at 24 hrs postoperative. The mean VAS score at two, four, and six hours and total rescue analgesic consumption in 24 hrs were higher in the control group, which was statistically significant (p-value <0.05). Quadriceps muscle strength by straight leg raise test was significantly higher in the Group ACB compared with the Group FNB at 0, 6, 12, 18 hours postoperatively (p-value <0.0001), whereas the difference between both study groups become statistically insignificant at 24 hours postoperative.

Conclusion: ACB preserved quadriceps muscle strength better than FNB, without a significant difference in postoperative analgesia after arthroscopic ACL tear repair surgeries.

## Introduction

Knee arthroscopic surgeries are being performed routinely for trauma, ligament injuries, meniscal injury, removal of synovial tissue, trimming damaged articular cartilages, knee sepsis, and patella problems. The surgery though considered minimally invasive is associated with significantly severe pain postoperatively which affects the quality of sleep, appetite and functionality of patients [1-4].

This pain needs to be attended to with utmost care and various pain-relieving modalities. Using peripheral nerve blocks (PNB) postoperatively as adjunct analgesia helps in attenuating postoperative pain and speeds the recovery of patients [5,6].

Effective pain management is essential after arthroscopic anterior cruciate ligament tear repair surgeries as it directly affects the patient’s pace of rehabilitation and recovery [7]. Therefore, postoperative pain management is essential for functional recovery and patient satisfaction. Careful planning to mitigate pain helps in early ambulation and physiotherapy, shortening hospital stays, earlier home discharges, and better patient satisfaction which reduces the burden on healthcare facilities.

PNBs can be easily performed by USG. Ultrasound guidance improves the success rate by shortening the block execution time, decreasing attempts and curtailing block onset time [8]. For adductor canal block (ACB), also named saphenous nerve block, a local anaesthetic is injected around the saphenous nerve, which lies directly anterior to the femoral artery at mid-thigh level [9]. The anterior articular branches of the femoral nerve are also blocked during the process and the majority of the anterior and medial areas of the knee joint are anaesthetized, which reduces pain in the intraoperative and early postoperative period [10]. For femoral nerve block (FNB), a local anaesthetic is injected around the femoral nerve in the inguinal canal. Motor branches of the femoral nerve that innervate the quadriceps muscle group are also blocked, which leads to decreased quadriceps muscle strength. Reduced quadriceps strength may increase the patient’s risk of falling when postoperatively initial ambulation starts [11]. So if a nerve is blocked distally to the quadriceps motor branches, it will provide comparable analgesia without reducing quadriceps strength that may help in early ambulation postoperatively [12].

The study was designed to compare the efficacy of ACB and FCB for postoperative analgesia and quadriceps muscle strength so as to achieve a better patient satisfaction after arthroscopic ACL tear repair surgeries.

## Materials and methods

This prospective, randomized, controlled and double-blind study was conducted at Shyam Shah Medical College, Rewa, Madhya Pradesh, India, between April 2019 and March 2020 after obtaining the Institutional Ethics Committee's approval (Approval number: 9465/SS/PG/MC/2019 dated May 23, 2019). The study was performed in accordance with the guidelines of the Declaration of Helsinki. Ninety adult patients with the American Society of Anaesthesiologists (ASA) grades I and II, posted for arthroscopic ACL tear repair surgeries under a subarachnoid block, were enrolled for the study. Patients who refused to give consent, had psychological disorders like language difficulty, mental illness, and dementia, and had significant systemic diseases like asthma, diabetes, hypertension, cardiovascular diseases, and allergy to local anaesthetic were excluded from the study. Considering an alpha error of 0.05 and power of study as 80%, the estimated sample size comes out to be 28 patients in each group. Therefore, we included 30 subjects in each group to replace any dropouts.

**Figure 1:**
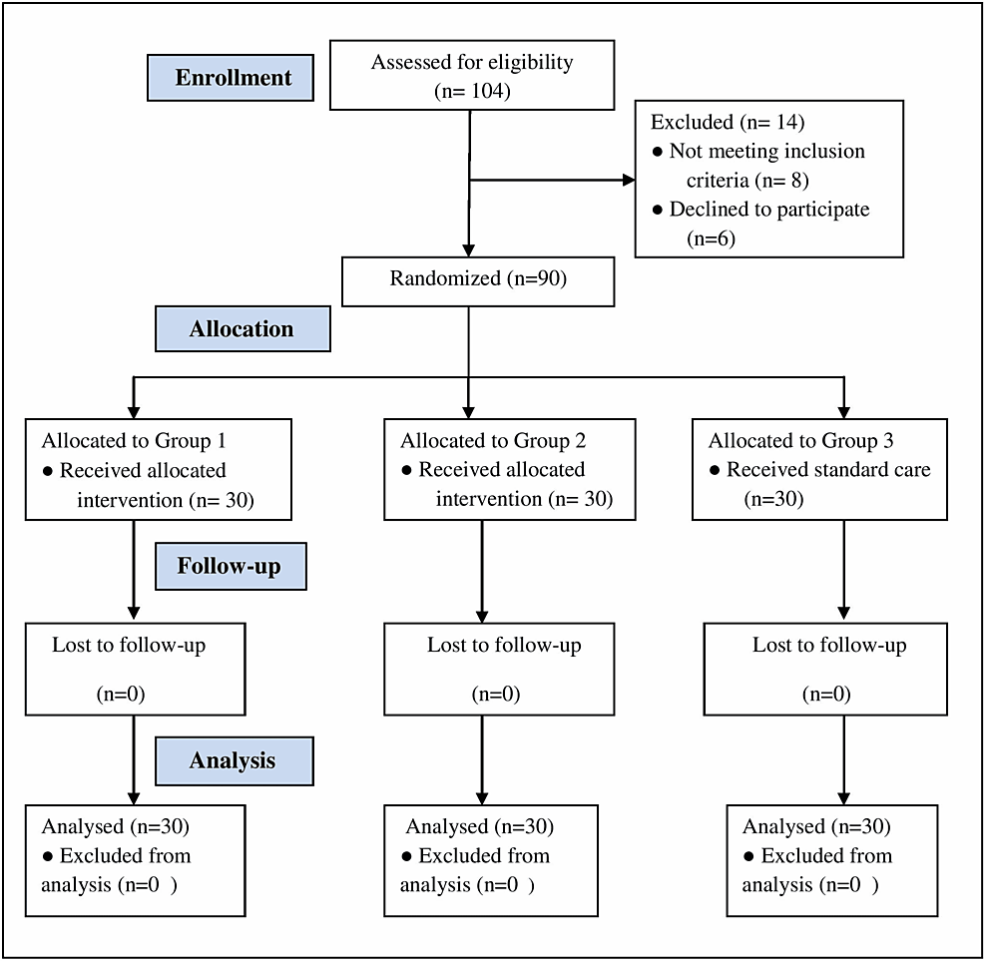
CONSORT flow diagram n: number of patients; CONSORT: Consolidated Standards of Reporting Trials

During the study, double-blinding was done to avoid bias. After randomization and group allocation, interventions were done by the principal investigator, observation by nursing staff, and statistical analysis by the statistician. Both nursing staff and statistician were unaware of the patient’s allocated group and the interventions performed. Patients fulfilling the selection criteria were randomized using computer-based randomization software in three groups, each of 30 patients (Figure 1).

Group 1 (Group ACB) received ACB with injection (inj.) of ropivacaine 0.25% 15 millilitres (ml) and inj. dexmedetomidine 0.5 microgram per kilogram (µg/kg) body weight, at the end of the surgery. Group 2 (Group FNB) received FNB with inj. ropivacaine 0.25% 15ml and inj. dexmedetomidine 0.5 µg/kg body weight, at the end of the surgery. Group 3 (Control group) received only the standard analgesic regimen.

A thorough pre-anaesthetic evaluation including the airway, back of the patient, and site of the block was done. The procedure was explained to the patients and informed consent was taken. The patients were educated about the Visual Analogue Scale (VAS) and the patient’s satisfaction scale.

In the operating room with all standard monitors, baseline parameters like heart rate, systolic and diastolic blood pressure, mean arterial pressure (MAP), oxygen saturation (SpO_2_), electrocardiogram (ECG) tracings were recorded. Subarachnoid block at L3-L4 space with inj. bupivacaine (0.5%) heavy was given to all patients in sitting position.

The ACB or FNB was performed under real-time USG guidance with a linear high-frequency ultrasound probe (Mindray DC-30, Mindray Medical International Limited, Shenzhen, China) at the end of the surgeries, in Group 1 and Group 2 respectively. As standard protocol, intravenous (IV) inj. diclofenac 75mg in 100ml normal saline and inj. paracetamol 1gm IV infusion was given to all the patients.

After completion of the surgery and block, patients were transferred to the post anaesthesia care unit (PACU). Pain severity was assessed by the PACU staff nurse employing a VAS. The VAS score was recorded at 0, 2, 4, 6, 8, 12, 18, and 24 hours after the block. When VAS score ˃4 or the patient first demanded analgesia, that time was noted and IV inj. tramadol 100 mg was given as rescue analgesic. At the end of 24 hours, the duration of analgesia and total tramadol consumption was noted.

Each patient was also assessed for quadriceps muscle strength. The patients (in supine position) were asked to raise the operated leg without flexing their knees (straight leg raise test). The motor block was graded as: Grade 0: normal muscle power; Grade 1: motor weakness; Grade 2: complete motor paralysis. The assessment for quadriceps muscle power was done in the postoperative care unit at 6, 12, 18, and 24 hours.

Any signs of adverse effects of the technique like infection at block site, hematoma formation, and/or local anaesthetic toxicity due to inadvertent intravascular injection (like dizziness, tinnitus, perioral numbness and tingling, seizures, signs of cardiac toxicity like arrhythmias, conduction block, myocardial depression) were also noted and managed accordingly. Neurological assessment was performed on all the patients before hospital discharge. The study ended 24 hours after block placement.

Statistical analysis

All recorded data were tabulated and statistically analyzed by appropriate statistical tests (ANOVA, Post hoc Tukey’s honestly significant difference (HSD) test, and Chi-square test). Continuous variables were presented as mean with SD and categorical variables were presented as frequency and percentages. Student’s t-test was used for testing the significance of the mean in both the groups. Qualitative data were analyzed using Chi-square test. All the statistical results were considered significant at p-value <0.05.

## Results

The demographic parameters like age (in years), height (in cms), and sex distribution were comparable between the groups (Table 1).

**Table 1:** Demographic characteristics

Parameters	Group 1	Group 2	Group 3	p-value			
				Group 1 vs. Group 2	Group 1 vs. Group 3	Group 2 vs. Group 3	
Age in years (Mean ± SD)	40±12	39±10	38±12	0.527	0.349		0.707
Height in cms (Mean ± SD)	162±8	160±8	162±7	0.332	0.919		0.373
Sex ratio (Male: Female)	24:6	25:5	23:7	0.812			

From Table 2 it is evident that duration of analgesia was longer in Group 1 and Group 2 as compared to Group 3 and the difference was statistically significant (p-value <0.05). Duration of analgesia was comparable in Group 1 and Group 2. Tramadol requirement was higher in Group 3 as compared to Group 1 and Group 2, which was statistically significant (p-value <0.05), while the difference between Group 1 and Group 2 was statistically comparable (Table 2).

**Table 2:** Duration of analgesia and total tramadol requirement * Statistically significant

	Group 1	Group 2	Group 3	p-value		
	Mean ± SD	Mean ± SD	Mean ± SD	Group 1 vs. Group 2	Group 1 vs. Group 3	Group 2 vs. Group 3
Duration of analgesia (hours)	11 ± 3	11 ± 3	5 ± 1	0.941	<0.0001*	<0.0001*
Total tramadol requirement (mg)	150 ±68.2	160 ± 67.5	223 ±63	0.570	<0.0001*	<0.0001*

**Figure 2:**
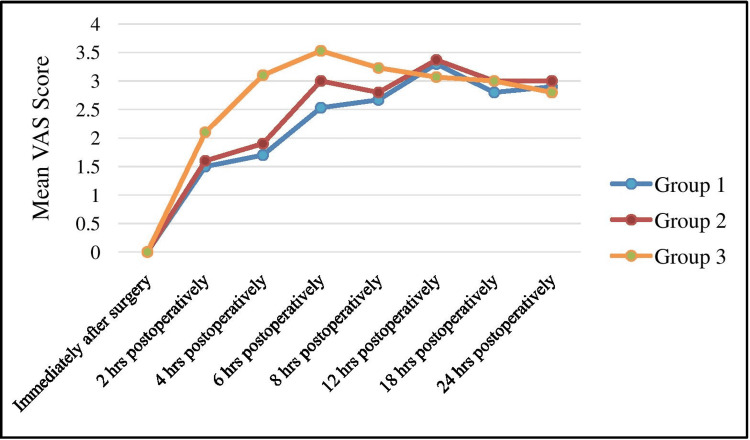
Graph showing mean VAS Score of Group 1 (ACB), Group 2 (FNB), and Group 3 (control) VAS- Visual Analogue Scale; ACB: adductor canal block; FNB: femoral nerve block

We found that the average of mean VAS score in first 24 hrs was 2.52±0.9 in Group 1, 2.61±0.91 in Group 2, and 2.94±1.03 in Group 3 (Table 3, Figure 2).

**Table 3:** VAS score at different time interval VAS: Visual Analogue Scale * Statistically significant

VAS Score	Group 1	Group 2	Group 3	p-value		
	Mean ±SD	Mean ±SD	Mean ±SD	Group 1 vs. Group 2	Group 1 vs. Group 3	Group 2 vs. Group 3
Immediately after the surgery	0	0	0	-	-	-
2 hours postoperatively	1.5±0.7	1.6±0.7	2.1±1	0.86	0.01*	0.03*
4 hours postoperatively	1.73 ±0.7	1.9 ±0.6	3.1 ±1.1	0.5285	<0.0001*	<0.0001*
6 hours postoperatively	2.53±0.86	3.00±0.95	3.53±0.90	0.1238	<0.0001*	0.0338*
8 hours postoperatively	2.67±0.9	2.8 ±0.9	3.23 ±0.7	0.58	0.02*	0.04*
12 hours postoperatively	3.3 ±1.12	3.37 ±1.1	3.07 ±0.91	0.817	0.379	0.253
18 hours postoperatively	2.8 ±0.9	3 ±0.9	3 ±1	0.569	0.403	0.786
24 hours postoperatively	2.9 ±0.9	3 ±0.9	2.8 ±0.71	0.668	0.632	0.348

**Figure 3:**
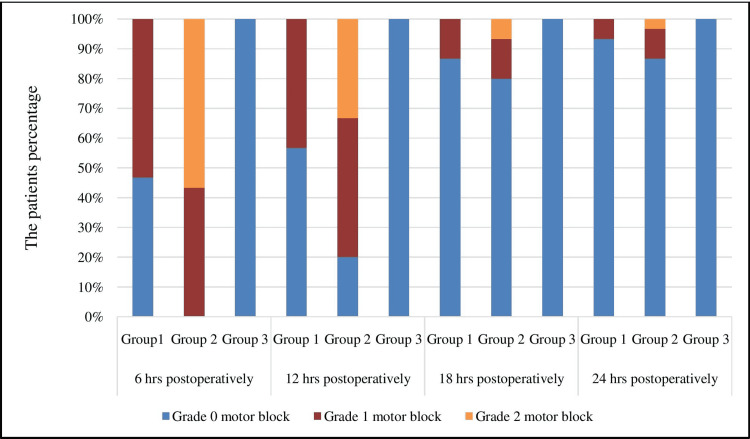
Graphs showing quadriceps muscle strength hrs: hours

Quadriceps muscle strength was assessed by the patients’ ability to perform a straight leg raise test. Assessment began six hours after surgery and then recorded at 12 hours, 18 hours, and 24 hours thereafter (Table 4, Figure 3).

**Table 4:** Quadriceps strength at different time intervals N: number of patients; %: percentage * Statistically significant

Quadriceps strength		Grade 0		Grade 1		Grade 2		Chi-square	p-value
		N	%	N	%	N	%		
6 hours postoperatively	Group 1	14	46.7	16	53.3	00	00	79.69	<0.0001*
	Group 2	00	00	13	43.3	17	56.7		
	Group 3	30	100	00	00	00	00		
12 hours postoperatively	Group 1	17	56.7	13	43.3	00	00	49.895	<0.0001*
	Group 2	6	20	14	46.7	10	33.3		
	Group 3	30	100	00	00	00	00		
18 hours postoperatively	Group 1	26	86.7	4	13.3	00	00	8.71	0.06
	Group 2	24	80	4	13.3	2	6.7		
	Group 3	30	100	00	00	00	00		
24 hours postoperatively	Group 1	28	93.3	2	6.7	00	00	5.08	0.28
	Group 2	26	86.7	3	10	1	3.3		
	Group 3	30	100	00	00	00	00		

We assessed the Patient’s Satisfaction Scale score 24 hrs after surgery, using a 5-Point Patient’s Satisfaction Score to evaluate the level of postoperative analgesia satisfaction (Table 5). The difference in the patient satisfaction score between groups was statistically significant (p-value <0.0001). There were no significant adverse effects or complications noted in any of the three groups.

**Table 5:** Patient satisfaction scale score N: number of patients; %: percentage of patients * Statistically significant

Patient Satisfaction Scale score	Group 1		Group 2		Group 3		p-value
	N	%	N	%	N	%	
Highly Satisfied	10	33.3	8	26.4	0	0	<0.0001*
Satisfied	11	36.7	9	30.3	6	20.0	
Neither Satisfied nor Dissatisfied	5	16.7	7	23.3	6	20.0	
Dissatisfied	4	13.3	6	20.0	14	46.7	
Highly Dissatisfied	0	0	0	0	4	13.3	
TOTAL	30	100	30	100	30	100	

## Discussion

Arthroscopic ACL reconstruction surgeries are associated with significant pain, which defies the aim of early mobilization and physiotherapy of patients. Various pain attenuating measures have been recommended like oral and parenteral analgesics, nerve blocks, infiltration of local anaesthetics, and patient-controlled analgesia.

In our study, we observed that the duration of analgesia was longer in groups receiving PNBs. Similar to our study, Faiaz et al. [13] noted that 25.7% of patients of group ACB and 29.3% of patients of group FNB need rescue analgesia and the difference was statistically comparable (p-value 0.730). Ghodki et al. showed a comparable time for the first rescue analgesic requirement in group ACB and group FNB, which was 12 hours (seven patients in group ACB and six patients in group FNB; p-value =0.75) [14]. Abdallah et al., in their study, observed a significant increase in time to introduce rescue analgesia in Group II (FNB) ((11.22±2.28) hours and Group III (ACB) (11.09±2.57) hours p-value 0.983) in comparison with Group I control (0) (p-value <0.001) [15]. The results of Lynch et al.'s study suggested similar effectiveness of ACB and FNB in postoperative pain control after ACL reconstruction [16].

The objective of our study was to render patients pain-free, with otherwise minimal effect on muscle strength. We found a statistically comparable difference in VAS scores among patients undergoing ACB and FNB, which shows similar analgesic effectiveness with both the techniques. Our study was in concordance with the observations of Bailey et al. [17], as they reported statistically comparable VAS at the different time intervals between group ACB (2.6±2) and group FNB (2.5±1.9) (p-value 0.793). Faiaz et al. also showed statistically comparable differences in VAS scores at postoperative 0, 12, and 24 hours; 2.29, 3.26 and 3.86 in group ACB and 2.59, 3.61 and 4.49 in group FNB ( p-value > 0.05) [13].

There were statistically significant differences in quadriceps strength between Group 1, Group 2 and Group 3 at 6 hours and 12 hours. Differences became statistically comparable between Group 1, Group 2, and Group 3 at 18 hours and 24 hours. The results of our study were similar to the study by El Ahl [18] as he observed significantly better quadriceps muscle strength in the ACB group at hours 0, 6, and 12 hours (p-value < 0.05), with statistically comparable differences at 18 and 24 hours (p-value > 0.05). Similarly, Abdallah et al., in their study, showed that ACB preserved quadriceps muscle strength in comparison to FNB in patients undergoing ACL reconstruction [19]. Edwards et al., in their study, demonstrated in patients undergoing ACL reconstruction, that ACB preserves quadriceps function better than FNB in the early postoperative period and simultaneously provides similar analgesia [20].

Our study showed that rescue analgesia requirement was lower in groups with nerve blocks than the control group. Our observation was similar to results found by Bailey et al. [17] that there was a statistically comparable difference between group ACB (16±7.4) mg and group FNB (14.8±8.3) mg for morphine consumption within 24 hours postoperatively (p-value 0.358).

Abdallah et al. also showed that the total morphine consumption was statistically comparable between group II (FNB) (1.37±3.87) mg and group III (ACB) (2.29± 4.78) mg (p-value 0.0943) but there was a significant higher analgesic requirement in group I control (14.11±4.63) mg in comparison to both group II and group III (p-value <0.001) [15]. Similarly, Kim et al. demonstrated a statistically comparable difference in opioid intake between the group FNB (50.4±33.1) mg and group ACB (50.3±30.8) mg (p-value 0.99) [21].

There are some limitations to our study. First is the non-inclusion of well-defined predictors of postoperative pain like preoperative anxiety and pre-existing pain conditions. Second, we did not measure postoperative hemodynamic vitals such as heart rate and blood pressure. Third, assessing patient satisfaction is quite subjective and is unavoidable to some extent. These limitations need to be addressed, and further multicenter studies with a large sample size may help provide data to overcome these shortcomings.

## Conclusions

According to the results obtained from our study, we observed that ACB and FNB both are better for pain relief from arthroscopic anterior cruciate ligament tear repair surgeries. ACB has shown better results when compared to FNB in terms of early ambulation and preservation of quadriceps muscle strength, which helped in early hospital discharge of patients. Hence, it is concluded that ACB is better than FNB for patients undergoing arthroscopic anterior cruciate ligament tear repair surgeries and is recommended.
